# The Temporal Associations of Therapeutic Alliance and Manual Adherence With Depressive Symptom Change in Cognitive Behavioral Therapy for Adult Outpatient Major Depression

**DOI:** 10.3389/fpsyt.2020.602294

**Published:** 2021-01-13

**Authors:** Frank J. Don, Ellen Driessen, Jaap Peen, Jan Spijker, Robert J. DeRubeis, Matthijs Blankers, Jack J. M. Dekker

**Affiliations:** ^1^Expert Center for Depression, Pro Persona Mental Health Care, Nijmegen, Netherlands; ^2^Department of Research, Arkin Mental Health Care, Amsterdam, Netherlands; ^3^Department of Clinical, Neuro and Developmental Psychology, Amsterdam Public Health Research Institute, Vrije Universiteit Amsterdam, Amsterdam, Netherlands; ^4^Department of Clinical Psychology, Behavioral Science Institute, Radboud University, Nijmegen, Netherlands; ^5^School of Arts and Sciences, University of Pennsylvania, Philadelphia, PA, United States

**Keywords:** therapeutic alliance, manual adherence, cognitive behavioral therapy, depression, temporal associations

## Abstract

**Background:** The therapeutic alliance is considered an important causal agent of psychotherapy efficacy. However, studies in cognitive behavioral therapy (CBT) for depression have suggested that alliance might be more of a consequence rather than a cause of depressive symptom change, while adherence to CBT specific techniques was found to be associated with subsequent depression change. We aimed to add to this body of literature by assessing the temporal associations of both therapeutic alliance and manual adherence with depressive symptom change in a relatively large sample of depressed adult outpatients over the full course of CBT.

**Methods:** Adults with a major depressive episode (*n* = 98) participating in a randomized clinical trial were offered 22 weeks of CBT and rated the Penn Helping Alliance Questionnaire (HAq-I) at weeks 5 and 22. Therapists rated their adherence to the CBT manual after each session and observers assessed the Hamilton Depression Rating Scale scores at weeks 0, 5, 10, and 22. Linear mixed model analyses were used to assess the associations of alliance and adherence with prior and subsequent depression change.

**Results:** HAq-I Relationship and manual adherence ratings were not significantly associated with prior nor with subsequent depression change (*p* > 0.14). Prior depression change was associated with the HAq-I subscale Perceived helpfulness at the end of treatment (*r* = 0.30, CI = 0.03–0.56, *p* = 0.03).

**Conclusion:** We were not able to replicate prior depression change in CBT for depression to be associated with improved quality of the therapeutic alliance when using a more “pure” measure of the therapeutic relationship. Limitations of this study include the subjective alliance and adherence assessments. Our findings indicate the need to appropriately distinguish between the perceived helpfulness and the relationship factors when examining therapeutic alliance.

## Introduction

Cognitive behavioral therapy (CBT) is one of the best-known and empirically supported psychological treatments for depression. Although CBT has shown to be efficacious in the treatment of depression (e.g., 1), still, up to 50% of patients fail to achieve an adequate response, and even fewer achieve remission following an acute treatment trial (1). Thus, the efficacy of CBT for depression needs to be improved.

One possible way to enhance efficacy is to investigate which treatment factors in CBT result in symptom change (2, 3), so that “we can direct better, stronger, different, or more strategies that trigger the critical change process(es)” (2). Two theories pose how CBT results in depressive symptom change: the behavioral activation theory (4) and the cognitive theory (5). Lewinsohn et al. (4) theorize that depression is caused or maintained by a reduction in satisfying activities. Patients are therefore encouraged to engage in activities that positively influence their mood. On the other hand, the cognitive theory posits that inaccurate beliefs and maladaptive information processing play a causal role in the development and maintenance of depressive symptoms (5). According to this theory, correcting these beliefs and processes is the core working mechanism in CBT for depression. In order to capitalize on these theorized effective treatment factors, it is important that therapies are being conducted as intended, i.e., that the therapist is adherent to the treatment manual (6, 7).

Besides the specific CBT factors mentioned previously, there is a more general—or non-specific—factor that is supposed to contribute to CBT efficacy. The quality of the therapeutic alliance is considered an important factor that can enhance the efficacy of psychotherapy in general (8, 9). According to Bordin's (10) influential definition, therapeutic alliance implies that therapist and patient (a) agree on treatment goals, (b) define a set of therapeutic tasks used to achieve the goals, and (c) form a positively toned emotional bond.

In an attempt to assess the associations of therapeutic alliance as well as therapist's adherence to the treatment manual with treatment effect, Castonguay et al. (11) found treatment adherence and the alliance—both as assessed at the end of treatment—to be associated with a better response to CBT for depression. However, in this study the temporal relation of adherence and alliance with symptom change was not adequately established (2). It did not rule out reverse causality: that it was symptom change that drove adherence and alliance rather than the other way around. Adequately taking reverse causality into account requires addressing the temporal relationship between treatment factors and outcome.

DeRubeis and Feeley (12) were the first to use this strategy when investigating process factors in CBT associated with alleviation of depressive symptoms. In a sample of 25 depressed outpatients, they found that the extent to which therapists adhered to the manual, i.e., they used concrete symptom-focused CBT in an early session, was associated with subsequent change in depression. This finding was replicated by Sasso et al. (13), Strunk et al. (14), and Brotman (15), and provides support for the behavioral activation and cognitive theories of depression. However, an investigation by Snippe et al. (16) among depressed diabetics following CBT failed to identify a significant association between adherence and treatment effect.

DeRubeis and Feeley (12) also found that therapeutic alliance was not correlated with subsequent change in depression, but was correlated with prior symptom change, suggesting that the quality of the therapeutic relation was more of a consequence rather than a cause of depression symptom change. This finding is particularly interesting, because it is contrary to the conventional wisdom that the quality of the therapeutic relation is an important causal agent of psychotherapy efficacy. It received only marginal support in a replication study by the same authors, where prior symptom change predicted therapeutic alliance at trend level only (17), but it was replicated in larger samples by Strunk et al. (14) and by Strunk et al. (18). However, the last two investigations were restricted to the first five and three sessions, respectively, leaving unanswered which changes may have taken place in later phases of treatment. Using a sophisticated repeated-measures design, Falkenström et al. (19) also found that improvement in alliance was associated with a reduction of depressive symptoms in the next CBT or IPT session, but the authors note the relatively small sample size as a limitation of their study (43 patients underwent CBT). Together, the findings of these studies suggest that in CBT for depression, quality of the therapeutic relation might be a consequence of depression symptom change. This is in contrast with studies investigating the alliance in treatments other than CBT for depression, which generally found the alliance associated with subsequent symptom change (20–28). It should be noted, however, that several studies also have failed to find a significant relationship between alliance and symptom change in CBT for depression (29, 30) even when applying a high-quality mediator study design. A meta-analysis among several kinds of psychotherapies and disorders by Flückiger et al. (31) revealed a significant relation only in investigations with a specific interest in the alliance.

In this study, we aim to add to the literature mentioned previously by investigating the temporal associations of both therapeutic alliance and manual adherence with depressive symptom change in CBT for adult outpatient major depression in a relatively large sample of patients over the entire course of the treatment. We hypothesize that prior symptom change will be positively associated with therapeutic alliance, but that therapeutic alliance will not be associated with subsequent symptom change. Furthermore, we hypothesize that early manual adherence will be associated with subsequent symptom change, but not with prior symptom change.

## Materials and Methods

### Design

This paper draws upon data from the CBT condition of a randomized clinical trial in the outpatient treatment of major depression (32). The study design was approved by the Dutch Union of Medical-Ethic Trial Committees for mental health organizations and the study protocol was published (33). Of the 341 participants randomized to treatment in the clinical trial, 164 were assigned to the CBT condition. Of these, 66 had a baseline HDRS score >24 and were offered additional antidepressant medication.

### Participants

Participants were referred by their general practitioner for depression treatment to one of three outpatient mental health clinics in Amsterdam, the Netherlands. Inclusion criteria were: (34) main diagnosis of depressive disorder according to DSM-IV criteria (35) as assessed by the MINI-International Neuropsychiatric Interview—Plus (36), (1) a score of 14 or above on the Hamilton Depression Rating Scale (HRSD, 30), (2) age between 18 and 65 years, and (3) a written informed consent after having received a complete description of the study.

Exclusion criteria included presence of psychotic symptoms or bipolar disorder, severe suicidality warranting immediate intensive treatment or hospitalization, substance misuse or abuse in the past 6 months, pregnancy, inability to meet trial demands due to for example medical conditions, and use of psychotropic or other medications that might influence mental functions. Patients on an antidepressant regimen were included only if the medication they were currently taking was judged to be inefficacious by both the patient and the intake psychiatrist. If so, the medication was tapered off under medical supervision, and baseline assessment took place after a washout period of at least 1 week after the medication was completely stopped. Patients with very severe depression (HDRS score >24) at baseline were offered additional antidepressant medication administrated by a psychiatrist. We excluded these patients for this work, because the effects of CBT could not be disentangled from those of the antidepressant medication.

Separate random allocation sequences were generated for each of the three clinics by one of the authors (J.P.) using SPSS random number generator (SPSS, Chicago). Randomization was stratified by gender and age (<32.5 and >32.5 years). Research assistants, aware of the allocation sequence, enrolled participants, and assigned them into interventions.

### Intervention

CBT comprised 16 individual sessions within 22 weeks, with the first 10 sessions taking place weekly and the final six taking place 2-weekly. CBT was conducted according to a published treatment manual (37) and consisted of an introductory session, three treatment phases and a concluding session. In the introductory session, acquaintance with the therapist was made, therapy conditions were explained, and a treatment contract was signed by both the patient and the therapist. The first treatment phase (sessions 2–4) focused on behavioral activation by means of planning and registering activities and concurrent mood levels. In the second CBT phase (sessions 5–7), the cognitive model was explained and patients kept a thought diary to identify automatic thoughts. These thoughts were challenged in the third phase (session 8–15), when they were tested on their validity and utility by logical reasoning. Patients were encouraged to identify reasoning errors in their own thinking. In addition, a behavioral experiment was designed and conducted to test the identified automatic thoughts in real life. Depending on the patient's needs, sessions 13–15 could be spent on complementary challenging techniques or conducting additional behavioral experiments. The final session (session 16) concluded treatment by evaluating the therapy and the therapeutic goals, and discussing strategies of action in case of relapse.

CBT therapists were psychiatrists or psychologists with at least a master's degree who completed a 100-h basic CBT training course accredited by the Dutch Association for Behavioral and Cognitive Therapy. Moreover, all therapists adequately conducted at least one intensively supervised therapy case in accordance with the treatment manual as judged by a study supervisor. Although no formal assessments were conducted, treatment fidelity was checked by means of biweekly supervision sessions, chaired by a study supervisor, in which audiotaped material was discussed. All study supervisors were registered supervisors with the Dutch Association for Behavioral and Cognitive Therapy.

### Measures

An overview of the assessments included in this work is provided in Table 1.

**Table 1:** Timeline of assessments during CBT treatment.

**Week**	**0**	**1**	**2**	**3**	**4**	**5**	**6**	**7**	**8**	**9**	**10**	**11**	**12**	**13**	**14**	**15**	**16**	**17**	**18**	**19**	**20**	**21**	**22**
Session		1	2	3	4	5	6	7	8	9	10		11		12		13		14		15		16
Depression severity	X					X					X												X
Therapeutic alliance						X																	X
Manual adherence		X	X	X	X	X	X	X	X	X	X		X		X		X		X		X		X

Depression severity was assessed with the Dutch version of the 17-item HRSD (38, 39) at weeks 0, 5, 10, and 22. The HRSD is a structured interview designed to quantify the severity of depressive symptoms in patients already diagnosed as suffering from a depressive disorder. Its items cover different depressive symptoms, such as mood, sleep problems, lack of appetite, weight loss, suicide intentions, and feelings of guilt, which are rated on either a 0–2 or 0–4 scale. Trained research assistants (master's-level graduate students in clinical psychology) assessed the HRSD according to the Dutch scoring manual (40). Assessors participated in biweekly 1-h peer supervision sessions, in which audiotaped interviews were discussed. The average intraclass correlation coefficient over 46 audiotaped assessments scored by multiple assessors was 0.97. The HRSD showed good reliability (Cronbach's alpha:0.82).

Therapeutic alliance was assessed from the patient's perspective at weeks 5 and 22 by means of the Penn Helping Alliance Questionnaire Method (HAq-I), which assesses the extent to which the patient experiences the therapist and the therapy as helpful (41, 42). The HAq-I is a self-report instrument including 11 items that are rated on a 6-point scale from −3 (“No, I strongly feel that it is not true”) to 3 (“Yes, I strongly feel that it is true”). The total score equals the sum of the item ratings. The HAq-I correlates well with other measures of therapeutic alliance (43–45) and the strength of the association between alliance and outcome assessed with HAq-I is comparable with other measures (46). The Penn Helping Alliance Scales distinguish two types of helping alliance. Helping Alliance Type 1 refers to the patient's perceived helpfulness of the therapist, whereas Helping Alliance Type 2 is defined as the patient's collaboration or bonding with the therapist. The HAq-I Type 2 subscale items have shown to form an independent factor (43, 47) that measures the collaborative nature of the therapeutic relationship. We used this subscale for the main analyses in this study, because, in our opinion, it better reflects Bordin's (10) definition of the therapeutic alliance. The reliability of both the total scores and Type 1 & 2 subscales of the HAq-I was good (Cronbach's alpha: type 1 alliance, 0.92; type 2 alliance, 0.92; total alliance 0.94).

CBT manual adherence was assessed by the therapist, who rated the extent to which he or she had been able to adhere to the treatment manual for that session on a scale from 1 (“not at all”) to 10 (“completely”) after each session.

### Data-analysis

We first calculated raw prior depression change scores for each patient by subtracting the HRSD score at week 0 from the HRSD scores at week 5, week 10, and week 22. Similarly, we calculated raw subsequent depression change scores by calculating the differences between the HRSD score at weeks 0, 5, and 10, and the end-of-treatment HRSD score at week 22. Next, we transformed the raw change scores to residualized change scores. More specifically, we used ANOVA to predict each patient's change based on their HRSD-score at week 0. We calculated the difference between the predicted score and the patient's actual change score resulting in a residual. A positive sign of this residual signifies a better-than-expected effect, while a negative sign indicates the effect is less than expected. We added the group mean change to the residuals and transformed these scores into z-scores to center the residual change around the group mean change (48)877–883.

With regard to therapeutic alliance, following Lorenzo-Luaces et al. (49), we used the sum score for the Relationship (Type 2)-subscale (items 1, 6, 7, 8, 9, and 10) for our main analyses, because this measures the collaborative nature of the therapeutic relationship, and not the perceived helpfulness that characterizes items from the Type 1-subscale. However, we also conducted sensitivity analyses using the HAQ-I total and the Type 1 (Perceived helpfulness) subscale sum scores.

With regard to manual adherence, mean adherence scores were calculated for sessions 1–5, sessions 6–10, and sessions 11–16, corresponding with treatment weeks 0–5, weeks 6–10, and weeks 11–22, respectively. We visually inspected Probability-Probability plots and judged all variables to be normally distributed.

We then assessed the associations of alliance and adherence with prior and subsequent depression change using linear mixed model analyses with a two-level structure (patient and therapist). In analyses of prior symptom change, alliance, or adherence served as the dependent variable and symptom change as the independent variable. In analyses of subsequent depression change, this variable served as the dependent variable and alliance or adherence as the independent variable. By design, alliance at week 22 (end of treatment) could only be related to prior depression change. Similarly, adherence in sessions 1–5 could only be related to subsequent symptom change and adherence in sessions 10–16 could only be related to prior symptom change.

In analyses including therapeutic alliance, the number of prior episodes was added as a covariate, because this variable has been found to moderate the alliance-outcome association in CBT for depression (49). In addition, we examined possible other confounders separately for each analysis, by testing whether the independent variable was significantly associated with one of the baseline characteristics (Table 2) using one-way ANOVA. As a result, we added gender as a covariate in the analysis of therapeutic alliance at week 5 and subsequent depression change (Table 3). Before computing the estimate of fixed effects, results were standardized into z-scores in order to get an *r*-type effect size following Strunk et al. (12, 730), where 0.20 represents a small effect 0.30 a medium sized effect and 0.50 representing a large effect (50). All analyses were conducted using SPSS version 22.0. The significance level used was alpha <0.05, 2-sided.

**Table 2:** Baseline characteristics of 98 patients assigned to cognitive behavioral therapy for depression.

	**M**	**SD**
Baseline HRSD	19.96	2.68
Age	37.29	10.79
	*n*	%
**Gender**		
Female	69	70.4
Male	29	29.6
**Marital status**		
Married	26	26.5
Divorced	16	16.3
Widowed	2	2.0
Never married	54	55.1
**Educational attainment**		
Low	18	18.3
Intermediate	36	36.7
High	42	42.9
Unknown	2	2.0
**Employment status**		
Employed	41	41.8
Student	4	4.1
Unemployed or “other”	53	54.1
**Episode duration**		
<6 months	33	33.7
6–12 months	22	22.4
1–2 years	14	14.3
+2 years	16	16.3
Unknown	13	13.2
**Prior episodes**		
0	35	35.7
1	16	16.3
2+	44	44.9
Unknown	3	3.1

**Table 3:** Associations of therapeutic alliance and manual adherence with prior and subsequent change in depression imputing all variables.

**Comparison**	***n***	***r***	***95% CI***	***p***
**Therapeutic alliance—Type 2 (Collaboration/Bonding)**				
Prior depression change—Therapeutic alliance week 5	56	0.14	−0.15 to 0.43	0.34
Prior depression change—Therapeutic alliance week 22	37	0.20	−0.07 to 0.47	0.14
Therapeutic alliance week 5—Subsequent depression change	48	0.14	−0.16 to 0.45	0.35
**Therapeutic alliance—Type 1 (Perceived helpfulness)**				
Prior depression change—Therapeutic alliance week 5	56	0.23	−0.05 to 0.52	0.11
Prior depression change—Therapeutic alliance week 22	37	0.30	0.03 to 0.56	0.03^*^
Therapeutic alliance week 5—Subsequent depression change	48	0.24	−0.01 to 0.50	*0.06*
**Therapeutic alliance—Total**				
Prior depression change—Therapeutic alliance week 5	56	0.20	−0.09 to 0.49	0.18
Prior depression change—Therapeutic alliance week 22	37	0.25	−0.01 to 0.54	*0.06*
Therapeutic alliance week 5 – Subsequent depression change	48	0.21	−0.08 to 0.49	0.15
**Manual adherence**				
Prior depression change—Manual adherence weeks 6–10	49	0.11	−0.19 to 0.41	0.47
Prior depression change—Manual adherence weeks 10–22	40	0.04	−0.22 to 0.31	0.75
Manual adherence weeks 1–5—Subsequent depression change	51	−0.05	−0.35 to 0.25	0.75

*N = 98*.

* *p < 0.05*.

Missing data for the different measures were imputed at item-level by means of multiple imputation, using the MICE package in “R” statistical software [version 2.25; (51)]. The default settings for the imputation method were applied, meaning that predictive mean matching was used for the imputation of missing numeric data, logistic regression imputation for binary data, polytomous regression imputation for unordered categorical data, and proportional odds model imputation for ordered categorical data. Variables with more than 50% missings were not imputed. Twenty imputed datasets were created. Density plots showed the distribution of the imputed data following the distribution of the original data, indicating adequate imputation. The analyses were performed on the 20 imputed datasets separately, and these results were combined using Rubin's rules (52) in SPSS. We used the imputed data for our main analyses, but we conducted sensitivity analyses using the observed (unimputed) data only. We also conducted sensitivity analyses in which the HRSD-scores at week 22 were not imputed but the other variables were, as imputation of the outcome variable has been disputed (53).

As an additional analysis we examined whether there was an interaction effect between the alliance at week 5 and manual adherence up to that point on subsequent symptom change in order to investigate whether manual adherence has more effect in the context of a good therapeutic alliance.

## Results

### Participants

From April 2006 to December 2009, 4,866 patients were assessed for eligibility during a standard intake procedure. Ninety-eight participants were included in the present study, 67 (69.8%) of which were female. Their mean age was 37.3 years (SD = 10.79; range 22–64) and their mean pre-treatment HRSD score was 19.96 (SD = 2.68), indicating severe levels of depression (54). The majority of the participants were never married (54.2%), had intermediate (36.5%) to high (42.7%) education levels, were unemployed (54.2%), had a depressive episode duration of <6 months (34.0%), and had reported two or more prior depressive episodes (46.2%). Detailed characteristics of the study sample are described in Table 2.

Thirty-five therapists treated on average 2.8 patients (range 1–9). The majority of patients had a female therapist (75.5%) with a mean age of 40.9 years (SD = 10.2, range 27–57). The average number of CBT sessions attended was 10.8 (SD = 5.5).

### Therapeutic Alliance and Symptom Change

The associations of therapeutic alliance with prior and subsequent depression change after imputation of missing data are shown in Table 3. Therapeutic alliance, as assessed with the Type 2 (Relationship) subscale at both week 5 (*r* = 0.14, CI: −0.15–0.43, *p* =0.34) and week 22 (*r* = 0.20, CI: −0.07 to 0.47, *p* = 0.14) was not associated with prior symptom change. Nor was therapeutic alliance Type 2 at week 5 associated with subsequent symptom change (*r* = 0.14, CI: −0.16 to 0.45, *p* = 0.35).

Sensitivity analyses with the Type 1 (Helpfulness) and HAq-I total sum scores also indicated no significant association between prior depression change and therapeutic alliance at week 5 (Table 3). However, prior symptom change was significantly associated with therapeutic alliance Type 1 at week 22 (*r* = 0.30, CI: 0.03–0.56, *p* = 0.03), and a similar association was found at the level of a non-significant trend for the HAq-I total score (*r* = 0.25, CI: −0.01 to 0.54, *p* = 0.06). HAq-Type 1 at week 5 predicted subsequent change at the level of a non-significant trend (*r* = 0.24, CI: −0.01 to 0.50, *p* = 0.06).

Sensitivity analyses using the observed data only and using the dataset in which all but the HRSD-score at week 22 were imputed are described in the (Supplementary Tables 1, 2). In the analysis of the unimputed data only, HAq-I Type 2 at week 22 was significantly associated with prior symptom change (*r* = 0.43, CI:0.13–0.68, *p* = 0.01), but not when imputing all variables but the HDRS at week 22 (*r* = 0.23, CI: −0.09 to 0.58, *p* = 0.15). We found no association between alliance at week 5 and prior or subsequent change in both sensitivity analyses (*p*s > 0.10), though HAq-I Type 2 at week 5 was associated with subsequent depression change at the level of a non-significant trend (*r* = 0.27, CI: −0.05 to 0.74, *p* = 0.08) when using the observed data.

### Manual Adherence and Symptom Change

The associations of CBT manual adherence with prior and subsequent depression change after imputation of missing data are shown in Table 3. Mean CBT manual adherence scores in the first 5 weeks of treatment were not associated with subsequent depressive symptom change (*r* = −0.05, CI: −2.36 to 1.70, *p* = 0.75). Mean adherence scores in weeks 5–10 and weeks 10–22 were not associated with prior symptom change up until these weeks either (5 and 10, respectively) (*r* = 0.11, CI: −0.19 to 0.41, *p* = 0.46 and = 0.04, CI: −0.22 to 0.31, *p* = 0.71). Sensitivity analyses using the observed data and the dataset in which all variables but the HRSD-scores at week 22 were imputed are described in the (Supplementary Tables 1, 2) and showed similar results. Again, no significant associations were found between manual adherence and prior or subsequent depressive symptom change (*p*s > 0.15).

There were no interaction effects between therapeutic alliance (HAq-I type 1, HAq-I type 2, and HAq-I total score) at week 5 and manual adherence up to that point on subsequent symptom change (*p*s> 0.05).

## Discussion

### Findings

We examined the temporal associations of therapeutic alliance and manual adherence with depressive symptom change in adult outpatients receiving CBT for depression. We found no association of CBT manual adherence with prior nor with subsequent symptom change. Similarly, we did not find therapeutic alliance to be related with prior nor with subsequent depression change in our primary analyses, in which we used the HAq-I type 2 subscale containing the items that purely tap the collaborative nature of the alliance. We did find prior depression change to be associated with the HAq-I type 1 subscale that assesses the perceived helpfulness, as well as for the total HAq-I scores (which is the sum of both subscales) at the level of a non-significant trend. We take this finding to indicate that patients who have experienced more depressive symptom alleviation over the course of their 22 week treatment also perceive their therapy and therapist as more helpful. Similarly, the perceived helpfulness subscale scores at week 5 were associated with subsequent depression change at the level of a non-significant trend, meaning that patients that perceive their therapist as helpful also may experience more symptom reduction.

Thus, we were not able to replicate previous studies finding prior symptom change to be associated with therapeutic alliance in CBT (12, 14) and CBT combined with antidepressant medication (18), when using a measure that, in our opinion, purely taps the collaborative nature of the alliance. Rather, our findings are in line with previous work in which no significant relationship between alliance and symptom change in CBT for depression was found (29, 30). We also did not find that the alliance is associated with subsequent depression change, which was also found in previous studies in CBT for depression (12, 14, 18), but not for other treatments like alliance fostering therapy (e.g., 15, 16) and supportive-expressive psychotherapy or clinical management combined with pharmacotherapy or clinical management combined placebo (27, 28). Maybe the therapeutic alliance plays a less important role in CBT for Depression than it does in other therapies (19). Concerning manual adherence, we also were not able to replicate prior studies finding adherence in CBT to be associated with subsequent depression change (e.g., 12, 50, 51), but our findings are in line with other work reporting no significant relation between adherence and treatment effect in CBT for depression (16).

### Strengths and Limitations

The study has a number of strengths. First, the study includes a relatively a large sample. Second, several elements contribute to the generalizability of the study's findings to general clinical practice. Treatment was provided in regular psychiatric outpatient clinics by a large number of therapists with different experience levels. Patients were not recruited by advertisement but instead were referred by general practitioners, no selection criteria with regard to previous treatment or suitability for psychotherapy were applied, and patients with relatively low socioeconomic status were included. Third, we carefully distinguished the collaborative nature of the therapeutic relationship and the perceived helpfulness of the therapist/therapy in our analyses. Fourth, our study design allowed us to examine the temporal relation between treatment effect and process variables and we used sophisticated statistical techniques to do so, controlling for patient and therapist variance.

This study also has a number of limitations. First, although depression symptom severity was assessed by independent observers, manual adherence and therapeutic alliance were subjectively assessed by, respectively, therapists and patients. Although the patient's perspective is frequently used in alliance research [e.g., (19, 55)], patients and therapists may be biased in their judgements by the improvement (or lack thereof) they experience. Independent raters, blind to outcome (56), may assess alliance and adherence more objectively. Second and related, symptom change and therapeutic alliance were rated later in treatment than in some other studies (e.g., 48) and we cannot rule out the possibility that an interaction between alliance and outcome might have already taken place at week 5. Third, although our research design allowed us to study the temporal associations of therapeutic alliance and manual adherence with symptom change, our design did not allow us to identify either of these variables as mechanisms of change (2). Neither does our study rule out possible third variable causality (that some unmeasured patient characteristic facilitated both the process variable and symptom change with no direct causal link between the two). Fourth, no control condition was included in the study.

### Clinical and Research Implications

The fact that we did not find a relation between treatment effect and the alliance does not mean that the alliance is irrelevant in CBT. It has been long suggested that in CBT for depression the alliance is necessary but not sufficient for therapeutic change to take place (e.g., 6). Rather, to put it in the words of DeRubeis and Feeley (1990), our well-trained and “empathetic therapists may undoubtedly have created a proper environment for therapeutic change.”

Concerning the limitations of this study, we recommend researchers investigating relations of alliance and adherence with symptom change to use more objective measures. Recent work has suggested that working mechanisms might be too complex to be captured in simple causal models (57) and this might also apply to the alliance and adherence. Indeed, for example Sasso et al. (58) found that protocol adherence has different aspects and may work differently in specific subgroups. Additionally, Lorenzo-Luaces et al. (49) found that the alliance-outcome association was moderated by the number of previous depressive episodes, also suggesting that the relationship between alliance and outcome can be different for different patients. We advocate further investigation of moderators of alliance-outcome and adherence-outcome relationships. Most importantly, however, our work underlines the importance of distinguishing the perceived helpfulness from the more pure relationship items of the HAq-I when examining therapeutic alliance and we recommend future investigations of therapeutic alliance to use an instrument not containing items that may also measure therapeutic progress as this can potentially bias results.

### Conclusions

We examined the temporal associations of therapeutic alliance and manual adherence with depressive symptom change in adult outpatients receiving CBT for depression. We found no association of CBT manual adherence with prior nor with subsequent symptom change. Similarly, we did not find therapeutic alliance to be related with prior nor with subsequent depression change in our primary analyses, in which we used the HAq-I type 2 subscale containing the items that purely tap the collaborative nature of the alliance. Thus, we were not able to replicate prior depression change in CBT for depression to be associated with improved quality of the therapeutic alliance. Our findings indicate the need to appropriately distinguish between perceived helpfulness and the more pure relationship items of the HAq-I when examining therapeutic alliance.

## Data Availability Statement

The dataset presented in this article can be accessed upon request. Requests can be directed to Jack J. M. Dekker, jack.dekker@arkin.nl.

## Ethics Statement

This study was reviewed and approved by Dutch Union of Medical Ethics Trial Committees for mental health organisations. The patients/participants provided their written informed consent to participate in this study.

## Author Contributions

FD and ED wrote the manuscript. ED coordinated the data acquisition. JP performed the statistical analyses. MB performed the imputations. RD contributed to the research design. JD an JS lead the research project. All authors provided comments and read and approved the final manuscript.

## Conflict of Interest

FD and JD receive fees from Springer Media. The remaining authors declare that the research was conducted in the absence of any commercial or financial relationships that could be construed as a potential conflict of interest.
