# Supplementary material for: The Temporal Associations of Therapeutic Alliance and Manual Adherence With Depressive Symptom Change in Cognitive Behavioral Therapy for Adult Outpatient Major Depression
Source: Front Psychiatry. 2021 Jan 13;11:602294. doi: 10.3389/fpsyt.2020.602294 (PMC7838346; doi:10.3389/fpsyt.2020.602294)
Supplement: Supplementary file 1 [file Data_Sheet_1.docx]

**Supplementary material**

**Table 1 Associations of therapeutic alliance and manual adherence with prior and subsequent change in depression - observed (unimputed) data.**

| **Comparison** | n | *r* | *95% CI* | *p* |
| --- | --- | --- | --- | --- |
| *Therapeutic alliance – Type 2 (Collaboration/Bonding)* |  |  |  |  |
| Prior depression change – Therapeutic alliance week 5 | 56 | .23 | -.06 to .65 | .10 |
| Prior depression change – Therapeutic alliance week 22 | 37 | .43 | .13 to .68 | .01* |
| Therapeutic alliance week 5 – Subsequent depression change | 48 | .27 | -.05 to .74 | .08 |
| *Therapeutic alliance – Type 1 (Perceived helpfulness)* |  |  |  |  |
| Prior depression change – Therapeutic alliance week 5 | 56 | .42 | .17 to .67 | .00 |
| Prior depression change – Therapeutic alliance week 22 | 37 | .72 | .48 to .95 | .00 |
| Therapeutic alliance week 5 – Subsequent depression change | 48 | .46 | .18 to .74 | .00 |
| *Therapeutic alliance - Total* |  |  |  |  |
| Prior depression change – Therapeutic alliance week 5 | 56 | .34 | .08 to .60 | .01 |
| Prior depression change – Therapeutic alliance week 22 | 37 | .58 | .33 to .83 | .00 |
| Therapeutic alliance week 5 – Subsequent depression change | 48 | .40 | .11 to .69 | .01 |
| *Manual adherence* |  |  |  |  |
| Prior depression change – Manual adherence weeks 6-10 | 49 | .19 | -.07 to .46 | .15 |
| Prior depression change – Manual adherence weeks 10-22 | 40 | .03 | -.29 to .36 | .83 |
| Manual adherence weeks 1-5 - Subsequent depression change | 51 | -.14 | -.43 to .13 | .29 |

^*)^ *^p^*^<.05^

**Table 2 Associations of therapeutic alliance and manual adherence with prior and subsequent change in depression – imputed data for all variables except HRSD at week 22.**

| **Comparison** | n | *r* | *95% CI* | *p* |
| --- | --- | --- | --- | --- |
| *Therapeutic alliance – Type 2 (Collaboration/Bonding)* |  |  |  |  |
| Prior depression change – Therapeutic alliance week 5 | 56 | .14 | -.24 to .59 | .40 |
| Prior depression change – Therapeutic alliance week 22 | 37 | .23 | -.09 to .58 | .15 |
| Therapeutic alliance week 5 – Subsequent depression change | 48 | .14 | -.25 to .54 | .45 |
| *Therapeutic alliance – Type 1 (Perceived helpfulness)* |  |  |  |  |
| Prior depression change – Therapeutic alliance week 5 | 56 | .25 | -.07 to .58 | .12 |
| Prior depression change – Therapeutic alliance week 22 | 37 | .33 | .04 to .61 | .03 |
| Therapeutic alliance week 5 – Subsequent depression change | 48 | .25 | -.07 to .56 | .13 |
| *Therapeutic alliance - Total* |  |  |  |  |
| Prior depression change – Therapeutic alliance week 5 | 56 | .21 | -.13 to .54 | .22 |
| Prior depression change – Therapeutic alliance week 22 | 37 | .29 | -.02 to 59 | .06 |
| Therapeutic alliance week 5 – Subsequent depression change | 48 | .20 | -.14 to .54 | .23 |
| *Manual adherence* |  |  |  |  |
| Prior depression change – Manual adherence weeks 6-10 | 49 | .10 | -.18 to .37 | .48 |
| Prior depression change – Manual adherence weeks 10-22 | 40 | .03 | -.23 to .30 | .81 |
| Manual adherence weeks 1-5 - Subsequent depression change | 51 | .00 | -.35 to .35 | .99 |

^*)^ *^p^*^<.05^
